# Detection of hydrogen cyanide from oral anaerobes by cavity ring down spectroscopy

**DOI:** 10.1038/srep22577

**Published:** 2016-03-04

**Authors:** Wen Chen, Kajsa Roslund, Christopher L. Fogarty, Pirkko J. Pussinen, Lauri Halonen, Per-Henrik Groop, Markus Metsälä, Markku Lehto

**Affiliations:** 1 Department of Chemistry, University of Helsinki, Finland; 2 Folkhälsan Institute of Genetics, Folkhälsan Research Center, Helsinki, Finland; 3 Abdominal Center Nephrology, University of Helsinki and Helsinki University Hospital, Helsinki, Finland; 4 Research Programs Unit, Diabetes and Obesity, University of Helsinki, Helsinki, Finland; 5 Oral and Maxillofacial Diseases, University of Helsinki and Helsinki University Hospital, Finland

## Abstract

Hydrogen cyanide (HCN) has been recognized as a potential biomarker for non-invasive diagnosis of *Pseudomonas aeruginosa* infection in the lung. However, the oral cavity is a dominant production site for exhaled HCN and this contribution can mask the HCN generated in the lung. It is thus important to understand the sources of HCN production in the oral cavity. By screening of oral anaerobes for HCN production, we observed that the genus of *Porphyromonas, Prevotella* and *Fusobacterium* generated low levels of HCN *in vitro.* This is the first study to show that oral anaerobes are capable of producing HCN *in vitro*. Further investigations were conducted on the species of *P. gingivalis* and we successfully detected HCN production (0.9–10.9 ppb) in the headspace of three *P. gingivalis* reference strains (ATCC 33277, W50 and OMG 434) and one clinical isolate. From *P. gingivalis* ATCC 33277 and W50, a strong correlation between HCN and CO_2_ concentrations (*r*_*s*_ = 0.89, *p* < 0.001) was observed, indicating that the HCN production of *P. gingivalis* might be connected with the bacterial metabolic activity. These results indicate that our setup could be widely applied to the screening of *in vitro* HCN production by both aerobic and anaerobic bacteria.

Hydrogen cyanide (HCN) is a toxic volatile compound capable of inhibiting cellular respiratory function. In nature, HCN can be found in plants and generated by bacteria. HCN is formed in seeds, roots and leaves of plants by hydrolysis of cyanogenic glycosides^1^. HCN is also released from bacteria through the metabolic pathway of bacterial cyanogenesis^2^. Bacterially produced HCN has been proven to be an effective virulence factor associated with growth suppression or killing of other living organisms^3^.To date, only a few bacteria are known to produce HCN: *Chromobacterium violaceum, Pseudomonas aeruginosa, Pseudomonas fluorescens, Rhizobium leguminosarum*^2^ and *Burkholderia cepacia*^4^. *B. cepacia* and *P. aeruginosa* are opportunist pathogens commonly associated with pulmonary infections in patients with cystic fibrosis (CF). Research has shown that *B. cepacia* was cyanogenic under biofilm growth on glass beads^4^, and elevated HCN levels were detected in the headspace of *B. cepacia* cultures^5^. *P. aeruginosa* on the other hand, is the main pathogen in the CF lung, and has been shown to produce gas phase HCN *in vitro*^6^.

*P. aeruginosa* infection can lead to chronic lung disease and eventual respiratory failure in patients with CF^7^, and therefore, it is important to detect its colonization at an early stage. Current diagnostic methods, including sputum sampling, bronchoalveolar lavage and blood antibody tests^8^, are invasive and can be difficult to perform, especially with children. It has been shown that the cultivation of *P. aeruginosa* from the sputum of CF patients generated notable amounts of HCN^9^, and that different strains of *P. aeruginosa* produce variable levels of HCN *in vitro*^6^. In addition, elevated levels of sputum cyanide were detected from *P. aeruginosa* infected CF patients, implying that *P. aeruginosa* generates HCN *in vivo*^10^,^11^. Since *P. aeruginosa* colonises in the lung and produces volatile HCN, it was assumed that exhaled breath HCN could be a potential biomarker to diagnose *Pseudomonas aeruginosa* infection non-invasively^12^. It has been reported that the concentration of mouth-exhaled HCN in children with *P. aeruginosa* infection (8.1–16.5 ppb) was significantly higher than those without the infection (0.0–4.8 ppb)^13^. A recent large-scale study confirmed mouth-exhaled HCN as a specific but insensitive biomarker of new *P. aeruginosa* infection in children with CF^14^. In addition to the HCN generated in the lung by *P. aeruginosa*, other sources contribute to the observed mouth-exhaled HCN levels. In adult *P. aeruginosa* infected patients, the concentrations of mouth-exhaled HCN (7.5–29 ppb) were not significantly elevated compared to healthy subjects (2.3–20 ppb)^15^. Studies in healthy volunteers have additionally shown that mouth-exhaled HCN levels are in general higher than nose-exhaled levels^16^,^17^. These findings indicate that there are significant sources of HCN within the oral cavity. Indeed, mouth-exhaled HCN levels of healthy subjects were correlated with HCN present in the oral fluid^18^. Furthermore, we have shown in a previous study that mouth-exhaled HCN levels decrease after a disinfectant mouth rinse^18^. These facts imply that a significant part of the mouth-exhaled HCN might be generated by oral bacteria.

Since HCN is a volatile metabolite of bacteria, it can be detected in the headspace of bacterial cultures. Headspace HCN measurements with selected ion flow tube mass spectrometry^19^ and photoacoustic spectroscopy^5^ have been applied to investigate HCN production by *P. aeruginosa*, which is an aerobic species. However, oral anaerobes have been found to play an important role in the production of breath volatiles^20^. Hence, it is essential to set up a novel headspace HCN sampling system for anaerobic bacteria.

The aim of our study is to set up a method to detect HCN from the headspace of both aerobic and anaerobic bacteria. Our apparatus is based on cavity ring down spectroscopy (CRDS). We screened four species of oral anaerobes for the production of HCN and conducted further investigations on four different strains of *P. gingivalis*. These *in vitro* measurements are the first steps towards elucidating the source of orally generated HCN.

## Results

### Method validation

**Figure 1:**
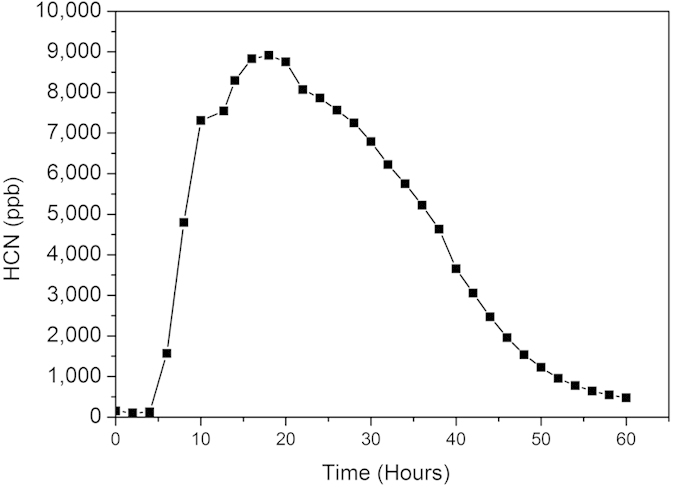
Dynamic profile of HCN production by *P. aeruginosa* ATCC BAA47. HCN concentration from the headspace of *P. aeruginosa* ATCC BAA47 was measured every 20 minutes. For clarity, data points (▪) are shown only every two hours.

*E. coli* ATCC 25922 and *P. aeruginosa* ATCC BAA47 were used as a negative and positive control, respectively, to show that our proposed system is valid for the determination of HCN from the headspace of bacterial cultures. HCN was not detected in the headspace of *E. coli* ATCC 25922 after 24 hours of culturing. In the headspace of *P. aeruginosa* ATCC BAA47, high level of HCN was detected after 12 hours of culturing. The HCN concentrations from triplicate samples were 4602, 3877 and 5207 ppb. Continuous online measurement was also tested using *P. aeruginosa* ATCC BAA47. Figure 1 shows the dynamic profile of HCN production by *P. aeruginosa* ATCC BAA47 during 60 hours of culturing. We observed that the concentration of HCN was low at the beginning of the culturing, but later on, the concentration increased dramatically and reached the maximum concentration of 8900 ppb at around 20 hours. After that, the HCN concentration dropped steadily until the end of the measurement (60 hours).

### Screening of HCN producing oral anaerobes *in vitro*

Seven strains of oral anaerobes were chosen for the screening test (Table 1). Five of them produced detectable amount of HCN in 72 hours, including *Porphyromonas gingivalis* ATCC 33277, *Porphyromonas endodontalis* ATCC 35406, *Prevotella intermedia* ATCC 25611, *Fusobacterium nucleatum subsp. nucleatum* ATCC 25586, and *Fusobacterium periodonticum* ATCC 33693. Since *P. gingivalis* ATCC 33277 produced the highest level of HCN among the five oral anaerobes, this strain was used in further investigations.

### Offline detection of HCN and CO_2_ produced by *P. gingivalis*

**Figure 2:**
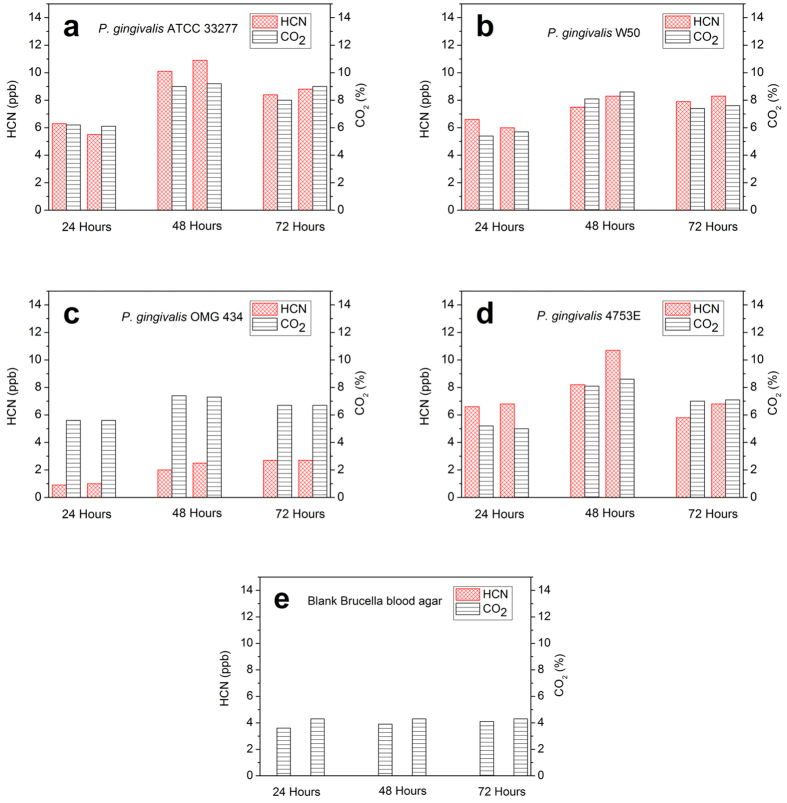
Detection of HCN and CO_2_ from *P. gingivalis* strains and blank Brucella blood agar. The *P. gingivalis* strains included three reference strains (**a**) ATCC 33277, (**b**) W50, (**c**) OMG 434 and one clinical isolate (**d**) 4753E. Blank Brucella blood agar (**e**) served as background control. There were duplicate samples for each strain. The detection was conducted at 24, 48 and 72 hours.

**Figure 3:**
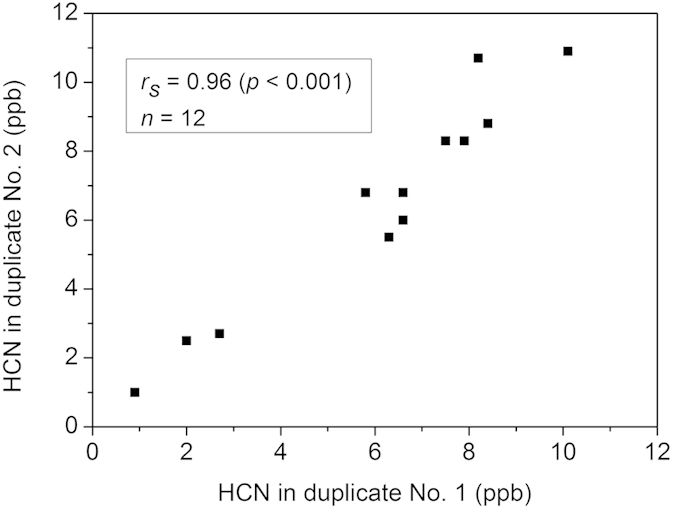
Correlation between duplicate samples in HCN offline headspace measurements of *P. gingivalis* strains. One data point (▪) represents the HCN concentrations obtained from duplicate No. 1 (horizontal axis) and No. 2 (vertical axis). Each strain was measured at 24, 48 and 72 hours, hence, there were three data points for each strain, and a total of 12 points for all four strains. A strong correlation (*r*_*s*_ = 0.96 and *p* < 0.001) was observed by Spearman’s rank correlation test.

HCN production from three reference strains (ATCC 33277, W50 and OMG 434) and one clinical isolate (4753E) of *P. gingivalis* were monitored (Fig. 2a–d). Blank Brucella blood agar, with hemin and vitamin K_1_, was used as a background control (Fig. 2e). All the samples were prepared as duplicates. In blank Brucella blood agar, the HCN level was lower than the instrumental detection limit (0.3 ppb), showing that the growth medium did not produce detectable levels of HCN. *P. gingivalis* ATCC 33277, W50 and OMG 434 represent three different serotypes A, B and C^21^, respectively. *P. gingivalis* ATCC 33277 produced 5.5–10.9 ppb of HCN during 72 hours of bacterial culturing. The highest concentration of HCN was detected at 48 hours. *P. gingivalis* W50 produced comparable HCN levels (6.0–8.3 ppb) to *P. gingivalis* ATCC 33277. The HCN levels detected from *P. gingivalis* OMG 434 (0.9–2.7 ppb) were much lower than those from *P. gingivalis* ATCC 33277 and W50. The clinical isolate *P. gingivalis* 4753E had similar HCN levels (5.8–10.7 ppb) compared to *P. gingivalis* ATCC 33277 and W50. CO_2_ concentration of 4% was detected from the blank Brucella blood agar, due to the anaerobic gas mixture (5% CO_2_, 10% H_2_ and 85% N_2_) used as the carrier gas in the measurements (Fig. 2e). The CO_2_ levels in the headspace of all *P. gingivalis* strains were higher than that of the blank agar. Since CO_2_ is one of the metabolites of *P. gingivalis*^22^, the elevated levels of CO_2_ indicate that all of the *P. gingivalis* strains were actively growing. In addition, the HCN levels from duplicate measurements were consistent with each other. A strong correlation (*r*_*s*_ = 0.96, *p* < 0.001) was observed between the HCN production of the duplicate samples of *P. gingivalis* strains (Fig. 3), implying that the HCN determination by our proposed system has high reproducibility.

### HCN to CO_2_ ratio

**Figure 4:**
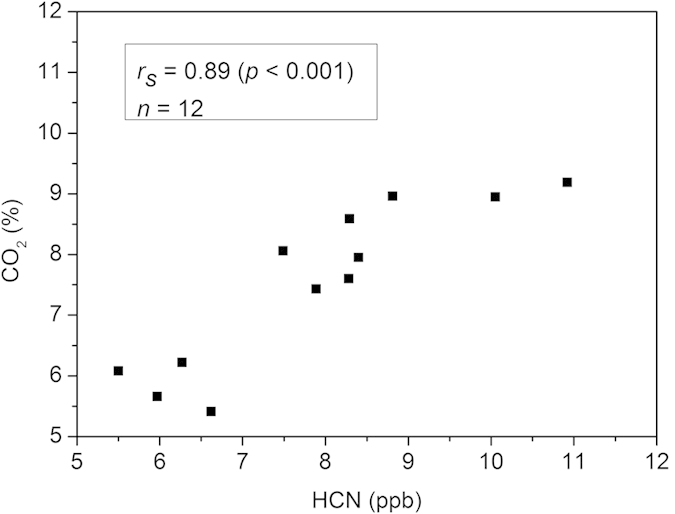
Correlation between HCN and CO_2_ concentrations of *P. gingivalis* ATCC 33277 and W50. Data points (▪) represent the concentration of HCN (horizontal axis) and CO_2_ (vertical axis) determined from one plate at a certain time point. Since each strain was measured in duplicate and at 24, 48 and 72 hours, each strain has six data points. In total, there are 12 data points for two reference strains, *P. gingivalis* ATCC 33277 and W50. A strong correlation (*r*_*s*_ = 0.89 and *p* < 0.001) was observed by Spearman’s rank correlation test.

The HCN to CO_2_ ratios of *P. gingivalis* ATCC 33277 (1.0 ± 0.1) and W50 (1.1 ± 0.1) were close to each other (Table 2). Coefficients of variation (CV) were low 10% and 9%, respectively. We further observed that there was a strong correlation between HCN and CO_2_ levels (*r*_*s*_ = 0.89, *p* < 0.001) in *P. gingivalis* ATCC 33277 and W50 (Fig. 4). Since the metabolite CO_2_ is an indicator of metabolic activity, these results imply that HCN production is also connected to the bacterial metabolic activity. However, with *P. gingivalis* OMG 434 the HCN to CO_2_ ratio was lower, and the CV was higher, than those of the other two reference strains. This is because *P. gingivalis* OMG 434 produced a lower concentration of HCN, which was close to the detection limit of our instrument. The clinical isolate *P. gingivalis* 4753E showed a similar HCN to CO_2_ ratio compared to *P. gingivalis* ATCC 33277 and W50, implying that the behaviour of the clinical isolate was similar.

### Online detection and dynamic profile of HCN production by *P. gingivalis*

**Figure 5:**
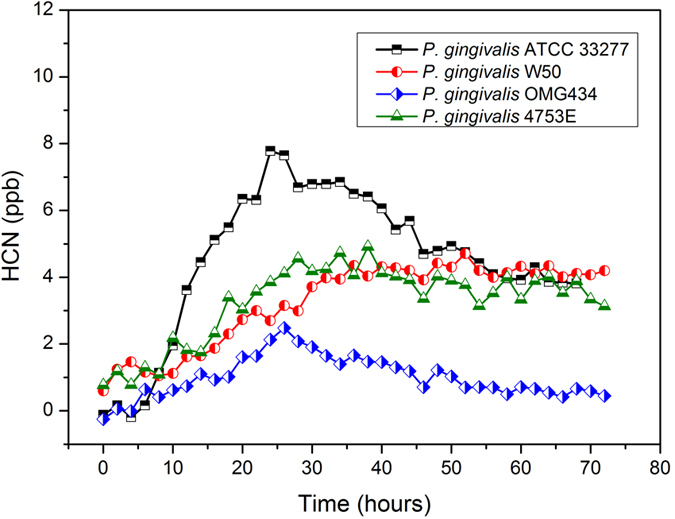
Dynamic profiles of HCN production by different *P. gingivalis* strains. The HCN concentrations from three reference strains (ATCC 33277, W50 and OMG 434) and one clinical isolate (4753E) of *P. gingivalis* were measured. The HCN concentrations from each strain were measured every 20 minutes. For clarity, data points are shown only every two hours.

The three reference strains of *P. gingivalis* had varying dynamic profiles of HCN production (Fig. 5). The HCN concentration of *P. gingivalis* ATCC 33277 increased steadily before reaching the maximum HCN concentration of 8.0 ppb within 20 hours, where after the HCN concentration dropped slowly until the end of the measurement. With *P. gingivalis* W50, the HCN concentration went up more slowly before reaching the maximum value of 4.5 ppb. No notable decrease in the HCN production was observed after the maximum, and the concentration kept stable till the end of the measurement. The third reference strain, *P. gingivalis* OMG 434, had a similar pattern of dynamic profile with *P. gingivalis* ATCC 33277. The maximum HCN concentration was around 2.5 ppb, which was the lowest among all the *P. gingivalis* strains. Furthermore, the clinical isolate, *P. gingivalis* 4753E, had a similar dynamic profile with *P. gingivalis* W50, i.e. HCN concentration increased slowly to maximum concentration of 5.0 ppb. At the end stage of the culturing, the HCN production remained stable at around 4.0 ppb.

## Discussion

Our study demonstrates that the proposed measurement setup can be used to detect HCN from the headspace of both aerobic and anaerobic bacteria. We successfully detected HCN emitted by the reference strain *P. aeruginosa* ATCC BAA47. The measured HCN concentrations were similar to the concentrations observed in previous studies^6^. A dynamic profile of HCN production by *P. aeruginosa* ATCC BAA47 was obtained by a real-time online measurement. The HCN concentration increased dramatically after a few hours, reached a maximum at around 20 hours and dropped steadily until the end of the measurement (60 hours). A similar pattern was detected previously by laser-based photoacoustic spectroscopy^5^.

In the screening test, we were surprised to find that five strains of oral anaerobes produced low but detectable levels of HCN. We showed that the genus of *Porphyromonas, Prevotella* and *Fusobacterium* are capable of producing HCN *in vitro. Porphyromonas gingivalis* ATCC 33277 produced the highest level of HCN among the oral anaerobes tested in this study. Based on these results, we further investigated the HCN production in the species of *P. gingivalis. P. gingivalis* is one of the well-known periodontal pathogenic microorganisms, which contribute to the development of periodontal diseases. Previous studies have shown that *P. gingivalis* produces different types of volatile metabolites, including volatile sulphur compounds (VSCs)^23^ and short-chain fatty acids^24^. However, none of the previous studies have demonstrated that *P. gingivalis* produces HCN. Three reference strains of *P. gingivalis* (ATCC 33277, W50 and OMG 434) were selected for this study, since they represent three different serotypes A, B and C, respectively^21^. In offline measurements, we found that ATCC 33277 and W50 produced similar concentrations of HCN, but OMG 434 produced much lower concentrations. Clinical isolate 4753E produced similar concentrations of HCN as ATCC 33277 and W50. This result indicates that the bacterial cyanogenesis in OMG 434 might be less active than in the other strains. All the strains produced detectable levels of HCN after 24 hours of bacterial culturing. At 48 hours, the level of HCN had increased with all strains. The HCN concentration detected at 72 hours was always lower or similar to that at 48 hours. According to these results, 48 hours of bacterial culturing is recommended in the screening of HCN production by *P. gingivalis* strains.

CO_2_ level was measured simultaneously with the HCN in the offline measurements. We observed that the CO_2_ levels of *P. gingivalis* were higher than those of the blank Brucella blood agar. It has been shown that CO_2_ is one of the metabolites in the metabolic pathway of *P. gingivalis*^22^. Hence, CO_2_ could serve as an indicator for metabolic activity of *P. gingivalis*. There was a strong correlation between HCN and CO_2_ produced by *P. gingivalis* ATCC 33277 and W50 (*r*_*s*_ = 0.89, *p* < 0.001). Although a significant correlation between HCN and CO_2_ was not found with *P. gingivalis* OMG 434 due to its low HCN concentration, we still observed an increase in the HCN level (from 0.97 to 2.25 ppb) as the CO_2_ level went up (from 5.6% to 7.4%). These results indicate that the metabolic activity of *P. gingivalis* has effect on its HCN production capacity, and confirm that HCN is one of the volatile metabolites of *P. gingivalis*.

To investigate the quantitative change in HCN production during the growth of *P. gingivalis*, we determined the dynamic profile of HCN production through online measurements. We found that *P. gingivalis* ATCC 33277 produced the highest amount of HCN, while *P. gingivalis* OMG 434 produced the least. This result is consistent with the observation from our offline measurements, indicating that both offline and online measurements are valid and reliable. We noticed that the HCN concentration detected in online measurement is generally lower than in offline measurement. There are two possible reasons for this. First, in the online measurements, there was a continuous anaerobic gas flow (10 mL/min), which flushed the bacterial container throughout the whole measurement. This might have a diluting effect on the HCN concentration produced by *P. gingivalis* strains. Second, during the offline measurements, both valves of the airtight container were shut at all times except when the samples were analysed. The HCN produced by *P. gingivalis* could be accumulated in the sealed container, resulting in a higher concentration of HCN. Bacterial growth is commonly evaluated by counting colony-forming units on microbial plates or using turbidity measurements in bacterial cultures. However, in our study it was not possible to determine the real-time growth curve for *P. gingivalis*, because the culturing was done on agar instead of broth. Although we could not show the effect of bacterial metabolic activity on HCN production by bacterial real-time growth curve, we could demonstrate this effect by the HCN to CO_2_ ratio as we discussed previously. In addition, the colony-forming units (CFU) were determined at the end of HCN measurements at 72 hours. The CFUs were in the range of 7.1 × 10^9^–3.9 × 10^10^.

Further studies are needed to clarify the underlying biological mechanisms, which regulate HCN biosynthesis in *P. gingivalis*. Identification of HCN synthase encoding gene cluster (*hcnABC*) from the genome of *P. gingivalis* would be one of the important tasks in future. Oral ecology might also affect the HCN production by *P. gingivalis in vivo.* Therefore, it will also be important to study the effects of co-culture with other oral anaerobes on the HCN production by *P. gingivalis*. HCN is considered as a potent virulence factor, which could increase tissue damage in host. Further studies are also needed to elucidate, whether HCN gas release by oral HCN-producing bacterial pathogens exacerbates the pathogenesis of periodontal diseases.

It is interesting to note that in addition to *P. aeruginosa*, large numbers of anaerobic bacteria have been detected in sputum samples of patients with CF, including species within the genus *Prevotella*^25^. The HCN levels measured for anaerobes in this study are much lower (<1%) than the levels measured for *P. aeruginosa in vitro*. Regardless, it might be worthwhile to investigate the effect of the anaerobes in the CF lung on the retrieved exhaled HCN levels of CF patients.

In conclusion, this study demonstrates that our proposed system can be applied to the screening of HCN production by both aerobic and anaerobic bacteria. To our knowledge, this is the first study to show that oral anaerobes generate HCN *in vitro*. Additionally, we found that HCN produced by *P. gingivalis* correlates significantly with the CO_2_ level, indicating that bacterial metabolic activity has an effect on HCN production. Further studies should be performed to search for an association between the existence of these cyanogenic anaerobes in the oral cavity of volunteers *in vivo* and their measured mouth-exhaled HCN levels. Additionally, *in vitro* studies should be conducted to try to optimize the culture conditions regarding the HCN production rate of the relevant anaerobes.

## Methods

### Bacterial strains and *in vitro* incubation conditions

The aerobic bacterial strains selected for this study were *E. coli* ATCC 25922 and *P. aeruginosa* ATCC BAA47. They served as a negative and positive control, respectively, for HCN headspace measurements. Both of strains were obtained from the American Type Culture Collection (ATCC). They were stored at −80 °C in frozen skim milk until analysis. These two aerobic bacteria were activated by streaking to tryptic soy agar (TSA) and cultivated at 37 °C for 24 hours. After the pre-culturing, a single colony was selected and streaked onto a new TSA plate. These plates were placed in airtight containers for the HCN headspace experiments. Two plates of *E. coli* ATCC 25922 and three plates of *P. aeruginosa* ATCC BAA47 were prepared for offline measurements. In addition, one plate of *P. aeruginosa* ATCC BAA47 was prepared for online measurement.

Seven strains of oral anaerobes for the screening test were *Porphyromonas gingivalis* ATCC 33277, *Porphyromonas endodontalis* ATCC 35406, *Prevotella nigrescens* ATCC 35563, *Prevotella intermedia* ATCC 25611, *Fusobacterium nucleatum subsp. nucleatum* ATCC 25586, *Fusobacterium periodonticum* ATCC 33693 and *Tannerella forsythia* ATCC 43037. To further investigate the HCN production by the species of *P. gingivalis*, three reference strains (ATCC 33277, W50 and OMG 434) and one clinical isolate (4753E) of *P. gingivalis* were selected. With the exception of OMG 434, which was from the Gothenburg (Sweden) Culture Collection, all other reference strains were obtained from ATCC. Clinical isolate 4753E was obtained from a patient with periodontal disease. All the strains were stored at −80 °C in frozen skim milk. They were activated by streaking onto Brucella blood agar (BBL^TM^ , 211086) plates, supplemented with horse blood (5% v/v), hemin (5 mg/L) and vitamin K_1_. Tryptic Soy Agar with n-acetylmuramic acid (TSA-NAM), supplemented with sheep blood, was applied to culture *T. forsythia* ATCC 43037. All the bacteria were incubated in anaerobic gas mixture (5% CO_2_, 10% H_2_ and 85% N_2_) at 37 °C for 72 hours. After the incubation, 5.0 mL of phosphate-buffered saline (PBS) was pipetted onto the Brucella blood agar plate. Bacteria were gently scraped from the agar and transferred into a Falcon^TM^ tube with the PBS. This bacterial suspension was homogenized by vortexing for 30 seconds. From this 5.0 mL of bacterial suspension, 0.1 mL was pipetted onto a new Brucella blood agar plate. This plate was placed in an airtight container for the HCN headspace experiment. For screening tests, one plate was prepared for each strain of oral anaerobes. For offline measurements, duplicate plates were prepared for each *P. gingivalis* strain. For online measurements, only one plate was prepared for each *P. gingivalis* strain. After the HCN headspace measurement, pour plate count was used to determine the colony-forming unit (CFU) of *P. gingivalis* on agar plates, to estimate their growth conditions. Different *P. gingivalis* strains had CFUs in the range of 7.1 × 10^9^–3.9 × 10^10^.

### Sampling system for aerobic bacteria

**Figure 6:**
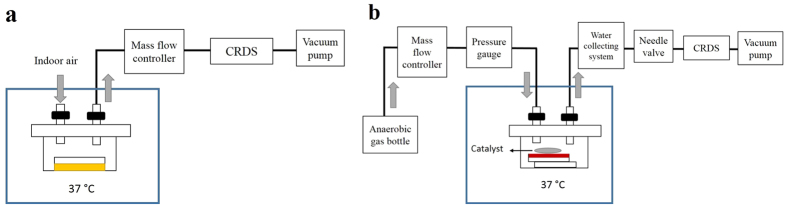
The sampling and measurement setup for HCN detection in the headspace of bacteria. (**a**) For aerobic bacteria indoor air was used as a carrier gas. The flow rate was controlled by a mass flow controller. (**b**) For anaerobic bacteria, anaerobic gas mixture (5% CO_2_, 10% H_2_ and 85% N_2_) was used as a carrier gas. Flow rate was controlled by mass flow controller. The pressure in the airtight container was monitored by pressure gauge and adjusted by a needle valve.

The experimental setup for HCN measurements with aerobic bacteria is shown in Fig. 6a. A Petri dish with the growth medium (tryptic soy agar, TSA) and the inoculated aerobic bacteria was placed into a 300 mL airtight container and kept at 37 °C. The airtight container had two valves, one of which was connected to indoor air. The other one was connected to a mass flow controller, which was linked to the CRDS instrument and a vacuum pump. The gas tubings were heated in order to maintain the temperature at 37 °C, and consequently, prevent condensation of the water produced by the bacteria.

In the HCN measurement for *E. coli* ATCC 25922, the Petri dish with the inoculated bacteria was placed into an airtight container and kept at 37 °C. Both valves of the container were open during the cultivation. After 24 hours of culturing, one of the valves was connected to a mass flow controller. An indoor air flow of 10 mL/min was applied in order to flush the headspace of the bacteria for 10 minutes. Consequently, the headspace containing the volatile metabolites transferred from the container into the CRDS instrument. The headspace measurement of *E. coli* ATCC 25922 was performed in duplicate.

In the measurement of HCN produced by *P. aeruginosa* ATCC BAA47, an indoor air flow of 50 mL/min was applied during the whole cultivation at 37 °C. For the first test, the HCN concentration from the headspace was determined at 12 hours. This measurement was performed in triplicate. Additionally, *P. aeruginosa* ATCC BAA47 was cultivated for 60 hours at 37 °C and the headspace HCN was measured and recorded at every 20 minutes, to obtain the dynamic profile of HCN production by the bacteria.

### Sampling system for anaerobic bacteria

The sampling system setup for HCN measurements with anaerobic bacteria is demonstrated in Fig. 6b. After the inoculation of oral anaerobes onto Brucella blood agar, the Petri dish with the growth media and bacteria was placed upside down on top of the Petri dish lid in an airtight container, to prevent water condensation on the agar. A catalyst was also put in the container to deplete oxygen. One valve of the container was connected to a pressure gauge, mass flow controller and anaerobic gas bottle (5% CO_2_, 10% H_2_ and 85% N_2_). The pressure gauge monitored the gas pressure inside the airtight container. The other valve was connected to a water collecting system, a needle valve, the CRDS instrument and a vacuum pump. The water collecting system prevented excess moisture, generated by the bacteria, from entering the measurement system. The needle valve was used for adjusting and stabilizing the gas pressure in the airtight container. The whole sampling system was first evacuated to 50 Torr. After this, the needle valve was closed and 350 mL/min anaerobic gas flow was introduced into the airtight container. Both valves of the container were shut, when the pressure display showed a pressure of 760 Torr. This step ensured that the container was at the atmospheric pressure. All the oral anaerobes were cultivated at 37 °C.

In the screening test and offline measurement, HCN concentration from the headspace of oral anaerobes were measured at 24, 48 and 72 hours. During the sampling, 10 mL/min anaerobic gas flow was applied to flush the container, and to transport the volatile metabolites into the CRDS instrument. After 10 to 15 minutes of air flow, the observed HCN concentration signal became stable. This indicated that the HCN concentration in the headspace and in the CRDS instrument were at equilibrium. At this point, the HCN concentration was measured. The screening test was performed once for each strain of oral anaerobes. The offline measurement of *P. gingivalis* was performed in duplicate. Two plates of blank Brucella blood agar were also measured in the offline measurement as background controls. In the online measurement, 10 mL/min anaerobic gas flow was applied continuously during the 72 hours of culturing. HCN concentration from the headspace of *P. gingivalis* was determined every 20 minutes to obtain the dynamic profile of HCN production.

### The CRDS instrument

CRDS was used for the detection and quantification of HCN in the gas phase. The spectrometer and data analysis procedures have been described in detail before^17^. The wavenumber scanning range for the measurement was 6504.2–6504.8 cm^−1^. This includes absorption peaks for HCN (6504.412 cm^−1^) and CO_2_ (6504.380 cm^−1^). The temperature of the sample cell was stabilized at 37 °C. The measurement pressure in the cell was 50 Torr. The estimated detection limit of the spectrometer for HCN in this study was 0.5 ppb. The absolute accuracy was confirmed by comparative measurements with a commercial reference HCN gas standard, which was certified to within 10%.

### Statistics

Spearman’s rank correlation test was used to analyze the correlation of HCN concentrations in duplicate measurements. This test was also used to analyze the correlation between HCN and CO_2_ concentrations of *P. gingivalis* ATCC 33277 and W50. In this test, the *p* value refers to the probability of obtaining the observation results assuming the correlation coefficient *r*_*s*_ is zero (null hypothesis).

## Additional Information

**How to cite this article**: Chen, W. *et al*. Detection of hydrogen cyanide from oral anaerobes by cavity ring down spectroscopy. *Sci. Rep.* **6**, 22577; doi: 10.1038/srep22577 (2016).

## Figures and Tables

**Table 1:** Screening of oral anaerobes for HCN production *in vitro.*

Oral anaerobes	HCN (ppb)		
	24 h	48 h	72 h
*Porphyromonas gingivalis* ATCC 33277	5.5	10.9	8.4
*Porphyromonas endodontalis* ATCC 35406	1.6	1.8	1.5
*Prevotella nigrescens* ATCC 35563	<0.5^a^	<0.5^a^	<0.5^a^
*Prevotella intermedia* ATCC 25611	3.9	4.0	3.6
*Fusobacterium nucleatum subsp. nucleatum* ATCC 25586	<0.5^a^	1.4	1.5
*Fusobacterium periodonticum* ATCC 33693	0.9	1.8	1.7
*Tannerella forsythia* ATCC 43037	<0.5^a^	<0.5^a^	<0.5^a^

^a^The HCN concentration lower than 0.5 ppb was below the detection limit of CRDS.

**Table 2:** HCN to CO_2_ ratio of *P. gingivalis* strains at 24, 48 and 72 hours.

*P. gingivalis*strains	HCN: CO_2_-ratio (ppb: %)			Mean ± s.d. (n = 6)	CV (%)^a^
	24 h	48 h	72 h		
ATCC 33277	0.96	1.16	1.02	1.0 ± 0.1	10
W50	1.14	0.95	1.08	1.1 ± 0.1	9
OMG 434	0.17	0.30	0.40	0.3 ± 0.1	39
4753E	1.32	1.13	0.90	1.1 ± 0.2	19

^a^The coefficient of variation (CV) is defined as the standard deviation divided by the mean value.
